# Leukoreduction and hydroxyurea in malignant pertussis: A case series

**DOI:** 10.1002/ccr3.3264

**Published:** 2020-08-25

**Authors:** Nedaa Aldairi, Hamza Alali, Yasser Kazzaz

**Affiliations:** ^1^ Department of Pediatrics Ministry of the National Guard ‐ Health Affairs Riyadh Saudi Arabia; ^2^ King Abdullah International Medical Research Center Riyadh Saudi Arabia; ^3^ Department of Pediatrics Collage of Medicine King Faisal University Al‐Ahsa Saudi Arabia; ^4^ College of Medicine King Saud bin Abdulaziz University for Health Sciences Riyadh Saudi Arabia

**Keywords:** *Bordetella pertussis*, hydroxyurea, leukoreduction, malignant pertussis, Saudi Arabia, whooping cough

## Abstract

We report five cases of malignant pertussis, a rare but life‐threatening illness. Current therapies include supportive care and leukoreduction. Notably, leukoreduction might be more effective when initiated prior to the development of organ failure and pulmonary hypertension.

## Graphical abstract

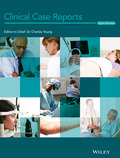

## 1 INTRODUCTION

Malignant pertussis is a rare, life‐threatening illness characterized by severe respiratory failure, severe leukocytosis, and pulmonary hypertension, with a high mortality rate.^1^ Current therapies include supportive care, leukoreduction, hydroxyurea, and extracorporeal membrane oxygenation. We report five confirmed pertussis cases with hyperleukocytosis. Three of the four patients who underwent leukapheresis died of cardiac arrest secondary to pulmonary hypertensive crisis. Early identification of risk factors and leukoreduction prior to the development of organ failure and pulmonary hypertension could be beneficial.

The coccobacillus *Bordetella pertussis* is re‐emerging after decades of control, despite being a disease preventable by vaccination.^2^ Pertussis is associated with multiple clinical syndromes in infants, including apnea/bradycardia pertussis, pneumonic pertussis, and malignant pertussis.^3^ For decades, leukocytosis has been known as a prominent characteristic of pertussis, correlating with disease severity and complications.^4^ This case series presents five patients admitted to the King Abdullah Specialized Children Hospital (KASCH), an academic governmental tertiary center in Riyadh, Saudi Arabia. Table 1 shows the patients’ demographics and characteristics, and the treatment provided.

**Table 1:** Summary of patients’ characteristics and provided therapy

	Case 1	Case 2	Case 3	Case 4	Case 5
Age	10 mo	7 mo	45 d	40 d	19 mo
Duration of symptoms	7 d	6 d	unknown	2 d	4 d
Immunization status	Unvaccinated	Unvaccinated	Ineligible for vaccine	Ineligible for vaccine	Unvaccinated
Initial WBC	77	74	123	32	110
Peak WBC	99	140	123	69	121
Intervention for Leukoreduction	Leukapheresis	Leukapheresis	Leukapheresis	Leukapheresis	Hydroxyurea
Inotropic support	None	Epinephrine and Dopamine	Epinephrine and norepinephrine	Epinephrine, Norepinephrine, Milrinone, and vasopressin	None
Ventilation	7 d	4 d	1 d	5 d	None
Nitric oxide	No	Yes	Yes	Yes	No
Echocardiography timeline (to PICU admission)	Day 2 of admission	First 24 h	First 24 h	Day 3 of admission	Day 3 of admission
Echocardiography finding	Tricuspid insufficiency, pressure gradient = 28 mm Hg, no echo signs of pulmonary hypertension	Dilated right atrium and right ventricle, Mild tricuspid valve insufficiency, RVSP 50	Moderate tricuspid regurgitation with estimated Pressure gradient 50 mm Hg, (RVSP 58, SBP 70)	Tricuspid regurgitation with gradient of 60 mm Hg and RVSP of 70 (SBP was 65 mm Hg, supra‐systemic pulmonary hypertension) with dilated right ventricle and D‐shaped left ventricle	Mild tricuspid valve insufficiency
Multi‐organ failure	No	Yes (CNS, liver, kidney, DIC)	Yes (CNS, liver, kidney, DIC)	Yes (liver, kidney, DIC)	No
Coinfection	None	Adenovirus and Bocavirus	Human Rhinovirus	None	Adenovirus, Human Rhinovirus, and Parainfluenza 3
PICU LOS	8 d	5 d	1 d	5 d	2 wk
Hospital LOS	23 d	10 d	1 d	5 d	47 d
Outcome	Recovery	Mortality	Mortality	Mortality	Recovery

Abbreviations: CNS, Central nervous system; DIC, Disseminated intravascular coagulation; LOS, Length of stay; PICU, Pediatric intensive care unit; RVSP, Right ventricle systolic pressure; SBP, Systolic blood pressure; WBC, white blood cell.

## 2 CASE SERIES

### 2.1 Case 1

A 10‐month‐old baby boy born preterm at 29 weeks, with a history of admission in the neonatal intensive care unit for respiratory distress syndrome, neonatal jaundice, and sepsis, was admitted to the pediatric intensive care unit (PICU) for bronchiolitis at 2 months of age. The baby was brought to the emergency room (ER) with a 1‐week history of cough and shortness of breath. Laboratory investigations revealed leukocytosis, and chest radiograph (CXR) showed diffuse inhomogeneous patchy opacity. The patient was not vaccinated for pertussis. Initially, azithromycin was administered for a differential diagnosis of pertussis, confirmed by respiratory‐panel polymerase chain reaction (PCR). Echocardiography did not indicate pulmonary hypertension.

During PICU admission, the white blood cell (WBC) count was 77 × 10^9^/L (72% lymphocytes and 16% neutrophils), and a peak count of 99 × 10^9^/L (65% lymphocytes and 24% neutrophils) was recorded without organ failure. On day 2, the PICU team discussed the hyperleukocytosis and decided to intubate electively for leukoreduction, after which the WBC count decreased to 38 × 10^9^/L. Following gradual pneumonia recovery after 7 days, the patient was extubated after appropriate weaning and discharged 2 weeks later (WBC count 15 × 10^9^/L during discharge).

### 2.2 Case 2

A 7‐month‐old unvaccinated boy, born after an uneventful full‐term pregnancy and perinatal course, was brought to the ER with cough and shortness of breath that had evolved over 6 days. There was positive history of sick contact. The patient was in mild respiratory distress and was clinically diagnosed with bronchiolitis. The initial WBC count was 17 × 10^9^/L, and the patient was hospitalized. After 5 days, he showed worsening respiratory distress and persistent tachycardia up to 180 beats/min. A partial septic screen revealed progressive leukocytosis with dominant lymphocytosis (WBC 73 × 10^9^/L; lymphocytes 41%, neutrophils 28%). The patient was transferred to the PICU and was given noninvasive ventilator support, but he had to be intubated after 2 days and required high ventilator settings and inotropic support with epinephrine. The WBC count progressively increased and peaked at 140 × 10^9^/L, and antibiotic therapy comprising of azithromycin, piperacillin/tazobactam, and vancomycin was administered. *B pertussis*, adenovirus, and bocavirus were confirmed by cultures and respiratory panel PCR. Laboratory tests showed persistent hyperleukocytosis, elevated liver enzymes, and coagulopathy, while echocardiography showed pulmonary hypertension.

The patient was switched to high‐frequency oscillatory ventilation (HFOV) and inhalational nitric oxide (20 ppm). After the first session of leukoreduction, the WBC count decreased from 140 × 10^9^/L to 60 × 10^9^/L, and transient clinical improvements in hypoxemia and hemodynamics were seen. However, he relapsed after 8 hours with rebound leukocytosis (100 × 10^9^/L). The second leukapheresis session decreased the WBC count to 47 × 10^9^/L; however, within a few hours, the patient experienced a pulmonary hypertensive crisis that progressed to cardiac arrest with no return of spontaneous circulation (ROSC).

### 2.3 Case 3

A 45‐day‐old baby girl was delivered after a full‐term uneventful pregnancy via emergency cesarean section due to failure to progress, with no significant perinatal events. She was brought to an academic medical center with limited facilities with complaints of cough and shortness of breath. The patient was admitted to PICU for apnea due to acute bronchiolitis. Her clinical condition remained unaltered with mild respiratory distress and hypoxia that required O_2_ supplementation. Laboratory investigations showed progressive leukocytosis, where the WBC count increased from 27 × 10^9^/L during admission to 82 × 10^9^/L with 60% lymphocytes after 24 hours. Based on a suspected diagnosis of pertussis pending laboratory confirmation, azithromycin was started. Echocardiography showed moderate tricuspid valve regurgitation with an estimated gradient of 50 mm Hg. The patient was transferred to our hospital with diagnosis of pertussis and hyperleukocytosis for further management and leukapheresis.

On transfer to the PICU, the patient was hypoxic and hemodynamically unstable. Epinephrine, norepinephrine, and milrinone were administered, along with HFOV. However, her condition progressively worsened to multi‐organ failure with acute kidney injury, encephalopathy, and coagulopathy. *B pertussis* and rhinovirus were confirmed with respiratory‐panel PCR. Broad‐spectrum antibiotics (vancomycin and ceftriaxone) were administered along with azithromycin considering an iatrogenic infection. The patient underwent leukapheresis, and the WBC count dropped from 123 × 10^9^/L to 37 × 10^9^/L. After 10 hours, there was rebound hyperleukocytosis (WBC count 100 × 10^9^/L), and another leukapheresis session was scheduled. However, cardiac arrest occurred after initiation of the second session, and cardiopulmonary resuscitation was undertaken without ROSC.

### 2.4 Case 4

A 40‐day‐old baby girl, born after a full‐term uneventful pregnancy, was brought to the ER with cough, cyanosis, and signs of mild respiratory distress. Vital signs at presentation included heart rate (HR) of 130 beats/min and 93% oxygen saturation. The patient developed frequent paroxysmal cough followed by apnea in the ER, and she was admitted to the PICU. The initial WBC count was 32 × 10^9^/L, and CXR showed bilateral patchy infiltration. Ampicillin and cefotaxime antibiotics were administered, but she had to be intubated because of repeated episodes of apnea and bradycardia. *B pertussis* was confirmed on respiratory‐panel PCR, and azithromycin was added to the antibiotic regimen. Echocardiography revealed tricuspid regurgitation with supra‐systemic pulmonary hypertension. The patient was placed on ionotropic support with epinephrine and milrinone, as well as HFOV with nitric oxide (20 ppm). However, her condition progressively worsened to multi‐organ failure with acute kidney injury, acute encephalopathy, acute liver injury, and disseminated intravascular coagulation. The WBC counts reduced from 69 × 10^9^/L (lymphocytes 63%, neutrophils 24%) to 19 × 10^9^/L after leukoreduction. Her respiratory status gradually worsened with signs of pulmonary hypertension. Hyperleukocytosis levels rebounded, and another leukapheresis session was performed, but pulmonary hypertensive crisis occurred that led to cardiac arrest without ROSC.

### 2.5 Case 5

A 19‐month‐old unvaccinated healthy girl, delivered via normal spontaneous vaginal delivery after a full‐term uneventful pregnancy, was brought to the ER by her parents for paroxysmal cough followed by cyanosis. The patient had been exposed to sick family members. She was hemodynamically stable (HR 130 beats/min), with signs of mild respiratory distress that necessitated administration of 1 L of O_2_ via nasal cannula. Laboratory investigations revealed a WBC count of 115 × 10^9^/L (45% lymphocytes and 35% neutrophils), and the patient was admitted to the PICU, where her WBC count ranged between 110 × 10^9^/L and 122 × 10^9^/L. Respiratory‐panel PCR confirmed *B pertussis*, adenovirus, human rhinovirus, and parainfluenza‐3 virus coinfection. Echocardiography did not reveal any signs of pulmonary hypertension.

The PICU, infectious disease, and hematology teams decided not to undertake leukapheresis in view of the patient's age and clinical stability. After 3 days of stable WBC count (109‐120 × 10^9^/L), hydroxyurea 20 mg/kg was administered, which was later increased to 30 mg/kg and administered as a single daily dose. The patient remained hemodynamically stable, and her leukocytosis decreased gradually. The patient was shifted to the ward, weaned off O_2_, and discharged after a 12‐day course of hydroxyurea (WBC count during discharge 36 × 10^9^/L).

## 3 DISCUSSION

Despite being a vaccine‐preventable disease with routinely available effective antibiotics, pertussis remains a major global concern and carries a significant risk of morbidity and mortality.^2^, ^5^ Hence, this mandates further research and resolution. Given the rarity of malignant pertussis and varied therapeutic options, a therapeutic trial might not be feasible. Therefore, reporting the clinical experiences of this disease is vital to shed light on the therapeutic management.

The surge in the global incidence of pertussis reflects disease awareness, advancement in diagnostic procedures, and possible changes in the *B pertussis* strain.^6^ Pertussis usually has three phases: catarrhal phase (1‐2 weeks), paroxysmal phase (2‐6 weeks), and convalescence.^2^, ^7^ Antibiotics and supportive respiratory care are the mainstay of pertussis management, regardless of the presenting syndrome.^2^, ^7^ Antibiotic treatment is undertaken to attenuate disease severity and shorten the duration of illness, which decreases its contiguity.^2^, ^7^ However, PICU admission might be needed, and this stage is considered critical in pertussis patients.^8^ Nonetheless, there is no defined standard clinical management for severe presentations of pertussis that are grouped under “malignant pertussis,” which is associated with higher mortality rates. Malignant pertussis is associated with respiratory failure, pulmonary hypertension, hyperleukocytosis, cardiogenic shock, and death.^1^, ^4^

The pathophysiological mechanism that mediates malignant pertussis is incompletely understood. A major part is attributed to the pertussis toxin (PT).^9^ The toxin‐mediated effect on the respiratory epithelium generates increased secretions and plugging, leading to increased pulmonary vascular resistance (PVR).^9^, ^10^ The PT triggers the frequently reported hyperleukocytosis causing sequestration, with or without thrombus formation, and induces rapidly progressing organ failure.^9^, ^11^ Moreover, PT acts in line with other virulent factors, and induces the release of nitric oxide and promotes cytopathologic effects,^12^ which is supported by the finding that inhaled nitric oxide acts as a mortality predictor in patients with pertussis.^12^, ^13^ Although physiologically sound, no alternative standard therapy for pertussis pulmonary hypertension exists. Accordingly, nitric oxide was used in majority of the reported cases in the literature as well as in three of our patients in this case series.^11^ All the aforementioned mechanisms have a common endpoint: a vicious cycle of ventilation‐perfusion mismatching and pulmonary hypertension that leads to intractable cardiopulmonary failure and death.^10^, ^11^, ^14^, ^15^

The invasive nature of the therapeutic options for malignant pertussis and lack of standardized management strategies makes it important to recognize the predictive outcome indicators early in the course of illness for appropriate management. Imminent risk factors include high or rapidly increasing WBC count (>50‐100 × 10^9^/L), persistent and consistent tachycardia, and high neutrophil/lymphocyte ratios.^14^ Other identified risk factors with variable correlation to morbidity and mortality include young infants (<2 months), seizure onset during illness, echocardiography findings of pulmonary hypertension, prematurity, and low birth weight.^4^, ^15^, ^16^

Hyperleukocytosis is considered a precursor to respiratory failure and pulmonary hypertension in pertussis; hence, leukoreduction may be a therapeutic modality to prevent disease progression and mortality.^9^, ^11^, ^17^ Literature regarding leukoreduction in malignant pertussis is limited, and it shows conflicting results. Rowlands et al recommended leukoreduction for pertussis hyperleukocytosis in their cohort of five patients who underwent leukoreduction and had pulmonary hypertension, of which four survived.^18^ Berger et al’s cohort did not show any clear therapeutic advantages with the various treatments conducted, including leukoreduction. Their cohort included 14 patients, of which eight developed pulmonary hypertension and five died, implying that further studies are necessary to examine the implications of leukoreduction.^8^

Leukoreduction is usually initiated based on a cut‐off WBC count (>50‐100 × 10^9^/L) and other risk factors.^18^, ^19^ In our cases, leukoreduction was successful in the hyperleukocytosis case, where leukapheresis was performed prior to the onset of pulmonary hypertension. On the other hand, leukapheresis was initiated after the development of pulmonary hypertension in the other cases, and none of the patients survived despite a reduction in the WBC count. Thus, leukoreduction initiation for hyperleukocytosis prior to the onset of pulmonary hypertension is worth exploring.

Leukoreduction or extracorporeal membrane oxygenation (ECMO) alone is insufficient to manage pulmonary hypertension, and a combination of both may be warranted.^20^ Moreover, ECMO might be considered in high‐risk patients with malignant pertussis who have already developed pulmonary hypertension. The cohort in the study by Rowlands et al reported survival of four out of five patients, and three of them underwent ECMO and leukoreduction sessions.^18^

Hydroxyurea, an antineoplastic agent that inhibits DNA synthesis, should be explored as a novel potential treatment for infection‐induced hyperleukocytosis in older infants and toddlers who seem to be at a lower risk of developing respiratory failure or pulmonary hypertension.^12^ The molecular mechanism of hydroxyurea action in malignant pertussis is based on theories of increased oxidative stress, which produces nitric oxide and alters blood cell adhesion to the endothelium. Maitre et al reported using hydroxyurea for malignant pertussis, showing clinical and biochemical responses.^10^

We simulated this approach in a toddler (Case 5) who was clinically stable to tolerate gradual WBC reduction and had asymptomatic pertussis‐induced hyperleukocytosis, in order to avoid invasive leukoreduction. The patient did not show respiratory failure or pulmonary hypertension. In our clinical experience, we observed a satisfactory albeit slow biochemical response. The WBC reduction occurred gradually over a 10‐14‐day period, which was reasonable given the clinical status of the patient.

Identifying poor prognostic factors (age, tachycardia, seizures, WBC count 50‐100 × 10^9^/L, and neutrophil/lymphocyte ratio > 1) early in pertussis helps build an index of suspicion for potential complications. More importantly, patients can be stratified into high‐ and low‐risk categories. In our series, age <2 months, elevated WBC count, and high neutrophil/lymphocyte ratio uniformly carried a higher probability of developing pulmonary hypertension, and eventually, death. We observed that a high neutrophil/lymphocyte ratio was associated with worsening of the disease course and mortality (Cases 2‐4, Table 2). This corroborates with a retrospective cohort study by Ganeshalingham A et al, wherein all children with malignant pertussis had neutrophil/lymphocyte ratios >1.^16^ Having any of these risk factors puts the patient in the high‐risk category, even before the appearance of any signs of organ dysfunction. This was evident in three of our patients, who progressed after a variable time gap of having the risk factors to multi‐organ failure, specifically acute kidney injury, acute encephalopathy, and disseminated intravascular coagulation with or without acute liver injury. Linking organ dysfunction directly to hyperleukocytosis needs autopsy confirmation, which was not performed in our cases.^11^, ^21^, ^22^

**Table 2:** Patients’ trends of WBC count, neutrophil‐lymphocyte ratio, and heart rate throughout their PICU stay

	Time 0	6 h	12 h	24 h	36 h	48 h	72 h	96 h	120 h	144 h
Case 1										
WBC	77		55	99	38	21	21	15	25	18
Neutrophil%	14		14	24	47	38	53		46	31
Lymphocyte%	72		78	65	42	40	27		32	42
N:L ratio	0.19		0.17	0.36	1.1	0.95	1.9		1.4	0.74
HR	140‐170	135‐165	118‐170	150‐190	150‐175	120‐170	120‐150	140‐180	110‐145	120‐140
Case 2										
WBC	74		87	120	119	132	140	100	69	
Neutrophil%	28		38	51	51	66	64	63	41	
Lymphocyte%	40		41	22	16	25	19	9	8	
N:L ratio	0.7		0.9	2.3	3.1	2.6	3.3	7	5.1	
HR	110‐150	120‐150	120‐160	150‐170	150‐190	160‐190	160‐190	170‐200	170‐ 190	0
Case 3										
WBC	123	43	37	59	49					
Neutrophil%	46	67	75	68	67					
Lymphocyte%	28	14	14	20	15					
N:L ratio	1.6	4.7	5.3	3.4	4.4					
HR	170‐220	190‐220	200‐220	200‐220	200‐220	0				
Case 4										
WBC	32		19	55	69	22	43	10	19	
Neutrophil%	22		20	31	24	49	45	30	55	
Lymphocyte%	69		70	62	63	27	17	35	15	
N:L ratio	0.32		0.28	0.5	0.38	1.8	2.6	0.8	3.6	
HR	120‐160	120‐150	42‐165	60‐165	130‐170	170‐185	165‐190	55‐195	200‐220	0
Case 5										
WBC	110		115	107	122	120	113	118	109	94
Neutrophil%	35		29	29	30	32	32	18	17	18
Lymphocyte%	45		61	54	61	53	62	73	75	73
N:L ratio	0.78		0.4	0.54	0.49	0.6	0.52	0.24	0.23	0.25
HR	145‐170	125‐165	115‐170	130‐150	110‐150	120‐160	110‐150	110‐160	110‐150	110‐155

Abbreviations: HR, Heart rate; N:L ratio, Neutrophil/lymphocyte ratio; WBC, White blood cell.

Evidence of risk factors should trigger close clinical and laboratory monitoring and early screening for pulmonary hypertension, immediately after recognizing the patient as high risk, using echocardiography to support an anticipatory management approach. Leukoreduction is an option in high‐risk patients. Careful observation with supportive respiratory care is reasonable for low‐risk patients. Hydroxyurea, an option for pertussis associated‐hyperleukocytosis, can be used for older infants and toddlers and as a “stop‐gap” during transfer and further intervention at another center.

In this case series, none of the patients who developed pulmonary hypertension survived, despite undergoing leukapheresis. ECMO was not offered to any patients. Hence, no recommendations based on this series can be made for the treatment modalities in pediatric malignant pertussis. Further reports exploring the hypothesized benefit of early initiation of leukoreduction prior to pulmonary hypertension initiation, and combination of ECMO and leukoreduction are needed.

In conclusion, close monitoring of HR, WBC count, and neutrophil/lymphocyte ratio is important for early recognition of children at a risk of malignant pertussis, and enable early PICU transfer for management with leukoreduction and ECMO. Due to the lack of evidence regarding the efficacy of leukoreduction or hydroxyurea, multidisciplinary discussion and local guidelines regarding treatment are recommended.

## CONFLICT OF INTEREST

The authors have no conflict of interest.

## AUTHOR CONTRIBUTIONS

Nedaa Aldairi: collected and analyzed the data, did the literature review, drafted the manuscript as submitted and agrees to be accountable for all aspects of the work. Hamza Alali: participated in collecting the data and critically reviewed the manuscript for important intellectual content. Yasser Kazzaz: participated in collecting the data and critically reviewed and supervised the writing of the manuscript.
